# Insulin-Stimulated Degradation of Apolipoprotein B100: Roles of Class II Phosphatidylinositol-3-Kinase and Autophagy

**DOI:** 10.1371/journal.pone.0057590

**Published:** 2013-03-13

**Authors:** Ursula Andreo, Liang Guo, Doru V. Chirieac, Ana C. Tuyama, Emilie Montenont, Jeffrey L. Brodsky, Edward A. Fisher

**Affiliations:** 1 Department of Medicine (Cardiology) and the Marc and Ruti Bell Program in Vascular Biology; and the Department of Cell Biology, New York University School of Medicine, New York, New York, United States of America; 2 Laboratory of Biochemical Genetics and Metabolism, Rockefeller University, New York, New York, United States of America; 3 Department of Biological Sciences, University of Pittsburgh, Pittsburgh, Pennsylvania, United States of America; University of Milan, Italy

## Abstract

Both in humans and animal models, an acute increase in plasma insulin levels, typically following meals, leads to transient depression of hepatic secretion of very low density lipoproteins (VLDL). One contributing mechanism for the decrease in VLDL secretion is enhanced degradation of apolipoprotein B100 (apoB100), which is required for VLDL formation. Unlike the degradation of nascent apoB100, which occurs in the endoplasmic reticulum (ER), insulin-stimulated apoB100 degradation occurs post-ER and is inhibited by pan-phosphatidylinositol (PI)3-kinase inhibitors. It is unclear, however, which of the three classes of PI3-kinases is required for insulin-stimulated apoB100 degradation, as well as the proteolytic machinery underlying this response. Class III PI3-kinase is not activated by insulin, but the other two classes are. By using a class I-specific inhibitor and siRNA to the major class II isoform in liver, we now show that it is class II PI3-kinase that is required for insulin-stimulated apoB100 degradation in primary mouse hepatocytes. Because the insulin-stimulated process resembles other examples of apoB100 post-ER proteolysis mediated by autophagy, we hypothesized that the effects of insulin in autophagy-deficient mouse primary hepatocytes would be attenuated. Indeed, apoB100 degradation in response to insulin was significantly impaired in two types of autophagy-deficient hepatocytes. Together, our data demonstrate that insulin-stimulated apoB100 degradation in the liver requires both class II PI3-kinase activity and autophagy.

## Introduction

ApoB100 is required in the liver for the assembly and secretion of very low density lipoproteins (VLDL), the precursors of low density lipoproteins (LDL). Both VLDL and LDL are atherogenic, and one of the statistically strongest risk factors for coronary artery disease is the plasma level of apoB100 (e.g., [1]). The fundamental importance of apoB100 to the biogenesis of VLDL and to atherosclerosis has stimulated considerable investigations on its metabolism. A major insight into apoB100 metabolism came in the mid-1980s with pioneering studies by Dr. Roger Davis, who used rat primary hepatocytes, and by Dr. Sven Olof-Olofsson, who employed human HepG2 cells [2], [3]. These investigators showed that a substantial amount of newly synthesized protein was subject to degradation. In contrast to most hepatic secretory proteins, in which the amount of secreted protein is tightly linked to the amount that is synthesized, variations in hepatic apoB100 and VLDL secretion were linked instead to the rate of apoB100 intracellular degradation (reviewed in [4]).

One of the metabolic signals that reduce VLDL secretion is insulin. For example, during acute hyperinsulinemia, such as occurs in the post-prandial state, hepatic VLDL triacylglyceride (TG) production becomes transiently depressed in humans (e.g., [5]) and in rodent models (e.g., [6]). It was proposed that one basis for this depression was insulin triggering a decrease in the amount of apoB100 that was available for VLDL assembly. Indeed, it was subsequently shown in model systems (typically, rodent primary hepatocytes) that an acute exposure to portal vein insulin levels (to simulate the post-prandial state) reduced cellular levels of apoB100, an effect largely attributable to its degradation (e.g., [7]).

It is now appreciated that the degradation of pre-secretory hepatic apoB100 occurs via at least two distinct pathways (reviewed in [4], [8]). One pathway is ER-associated degradation (ERAD), which requires the ubiquitin-proteasome system. During this process, nascent apoB100 that is insufficiently lipidated during its translation and translocation is recognized by cytoplasmic and ER lumenal molecular chaperones, which then deliver the protein for ubiquitinylation and degradation. The second pathway has been termed post-ER, pre-secretory proteolysis (PERPP), and was first described in studies on the effects of fish oil fatty acids on hepatic apoB100 and VLDL production [9]. In this case, and in two other examples of apoB100 PERPP (human apoB100 mutant A31P and glucosamine-induced apoB100 degradation), evidence has been presented to implicate autophagy as the culprit responsible for the proteolytic process [10]–[12]. Because insulin-induced apoB100 degradation is also a post-ER process [13], we hypothesized that insulin also directs apoB100 for degradation via autophagy.

In this report, we provide data using primary mouse hepatocytes to support this hypothesis. Furthermore, because PI3-kinases can regulate autophagy [14]–[16], and because insulin-mediated apoB100 degradation is compromised by non-specific PI3-kinase inhibitors, such as wortmannin (e.g., [17]), we also sought to determine which specific PI3-kinase class triggered the autophagic destruction of apoB100. By using a variety of methods, we have established that the class II PI3-kinase mediates this event. Besides providing new insights into the fundamental processes of VLDL assembly and secretion, our data also provide a molecular basis for the observed increase in apoB100 and VLDL production in insulin-resistant individuals, and suggest future therapeutic avenues to overcome the accompanying risk of cardiovascular disease in these individuals.

## Materials and Methods

### Reagents

Protease inhibitor cocktail tablets were obtained from Roche (Indianapolis, IN) or Sigma-Aldrich (St. Louis, MO). Protein A - Sepharose was obtained from GE Healthcare (Uppsala, Sweden) or Invitrogen (Frederick, MD). [^35^S]-methionine/cysteine protein labeling mix was obtained from Perkin Elmer Life Sciences (Waltham, MA). Goat anti-mouse apoB polyclonal antibody was a gift of Dr. Kevin J. Williams (Department of Medicine, Temple University) and was previously characterized [18]. The PI3-kinase inhibitor wortmannin was purchased from Sigma-Aldrich (St. Louis, MO). The class I-specific PI3-kinase inhibitor PIK-75 was purchased from Cayman Chemical (Ann Arbor, MI). The insulin was from Sigma-Aldrich (St. Louis, MO).

### Animals and primary hepatocyte culture

All animal procedures were approved by the Institutional Animal Care and Use Committee, NYU School of Medicine.

Mice deficient in APOBEC1 (*Apobec1^−/−^*) on a C57BL6 background were provided by Dr. Janet Sparks (University of Rochester) with permission from Dr. Nicholas Davidson (Washington University; [19]). Mice normally synthesize both apoB100 and apoB48 in their livers. The deletion of the apoB mRNA editing enzyme 1 (APOBEC1), which generates a mRNA encoding apoB48 from the apoB100 transcript, results in the production of only apoB100, the form secreted by human liver [20], [21]. Atg5 is an essential autophagy factor [14]. *Atg5*-floxed mice [22] were given to us by Dr. Steven Burden (NYU School of Medicine) with permission from Dr. Noboru Mizushima (Tokyo Medical and Dental University) and crossed with *Alb-Cre* mice (mice with the Cre-recombinase gene driven by the albumin promoter; Jackson Laboratory}) to generate liver specific *Atg5*-deficient mice. The genotyping of the *Atg5*-floxed mice was performed as previously described [23]. The genotyping of *Alb-Cre* mice was performed according to the protocol described by the Jackson Laboratory. In some experiments, hepatocytes were isolated from mice with either the floxed or the deleted *Atg5* allele that were crossed with *Apobec1^−/−^* mice.

Mouse primary hepatocytes were prepared by perfusion of livers with collagenase I (Worthington Biochemical) or Liberase TM (Roche). Hepatocytes were maintained in Waymouth’s medium (Waymouth’s MB 752/1, containing 1% streptomycin/penicillin, 1% L-glutamine, 0.2% BSA, and 0.1 nM insulin). After a 12–14 h incubation, the primary hepatocytes were subjected to study protocols.

### Pulse-chase metabolic labeling experiments

Portal levels of post-prandial insulin can achieve concentrations in excess of 100 nM. After pilot dose-response studies, protocols were designed so that primary hepatocytes were treated with 100 nM of insulin. In some experiments, there was co-treatment with wortmannin (100 nM) or the class I PI3-kinase specific inhibitor PIK75 (100 nM; [24]). The various treatment media were applied to the cells, which were then pulse-labeled with 240 µCi/ml [^35^S]-protein labeling mix for 15 min, and chased for 30 min and 120 min in non-radioactive medium with excess methionine (1.5 mg/ml) and cysteine (0.5 mg/ml). The first chase point was chosen according to the difference in apoB100 translation efficiency in different hepatic cells [10], [25]. The metabolic treatments were continued in the chase period. At the end of the chase, cells and conditioned medium were collected. [^35^S]-labeled apoB100 from cell lysate and conditioned medium was immunoprecipitated, separated by 4% SDS-PAGE, and then visualized using a phosphorImager (Trio Typhoon; Amersham, GE healthcare), with quantification by densitometry of the apoB100 bands. Total labeled apoB100 protein recovery was normalized to the amount of total labeled protein that was precipitated with trichloroacetic acid (TCA).

### PI(3,4)P_2_ quantification

Cellular PI(3,4)P_2_ concentrations were measured by using anti-PI(3,4)P_2_ mouse monoclonal antibody (Echelon Biosciences) following manufacturer’s instructions. Briefly, cells were collected in ice-cold 0.5 M TCA, and the pellet was washed in 5% TCA with 1.0 mM EDTA. Neutral lipids and acidic lipids were extracted sequentially by adding MeOH:CHCl_3_ (2:1) and MeOH:CHCl_3_:12 M HCl (80:40:1), and acidic lipids were collected by phase separation in 0.75 mL of CHCl_3_ and 1.35 mL of 0.1 M HCl. PI(3,4)P_2_ was quantified by dot blotting using anti-PI(3,4)P_2_ mouse monoclonal antibody.

### siRNA treatment of hepatic cells

For siRNA experiments, cells were treated for 48 h with SiGenome® pools (for control siRNAs or ones specific for mouse PI3-kinase CII gamma or PI3-kinase CIII (Vps34); Dharmacon, Thermo Fisher Scientific) and the DharmaFECT 4 transfection reagent (Dharmacon, Thermo Fisher Scientific) according to the manufacturer’s protocol. Pulse-chase experiments were performed as described above. The efficiency of PI3-kinase knockdown in mouse primary hepatocytes was assessed by a two step RT-PCR protocol using the ABsolute Blue SYBR Green Mix from Thermo Scientific and the following primers: ATTTCCTCACTGGTGGCATC (forward) and GAGCCTCGAGTCCTCATACG (reverse) for PI3-kinase CII gamma, CGAGGGAGCAGGAACATCA (forward) and TGTGCGGCATAGACAGTGAAC (reverse) for PI3-kinase CII alpha, CCCTCGAACCTATACTTCTCGATATG (forward) and TGGCCATTGGCAACAGTGT (reverse) for PI3-kinase CII beta, and GGCACCCAGAGTGAGCAGTAC (forward) and CAGGTGGAGGAAGGCTGTGT (reverse) for PI3-kinase CIII .

### LC3 Western Blot

Primary hepatocytes were prepared as described previously [22]. Total protein was extracted using RIPA buffer (50 mM Tris, pH 7.4, 150 mM sodium chloride, 0.25% sodium deoxycholate, 1% Nonidet P-40) and 50 µg was used for Western Blot analysis using rabbit polyclonal anti-LC3 antibody (dilution 1/500) from Novus Biological. Mouse monoclonal anti-GAPDH antibody (dilution 1/5000) was used as a loading control (Millipore).

### Lipoprotein fractionation

Primary hepatocytes were radiolabeled with 100 µCi/ml [^35^S]-Met/Cys for 4 h. Lipoproteins in the conditioned medium were separated by cumulative rate flotation (density gradient) ultracentrifugation. Briefly, 4 ml of the sample, adjusted to d = 1.10 g/ml with solid KBr, were overlaid with 3 ml of d = 1.065 g/ml KBr, 3 ml of d = 1.02 g/ml KBr, and 3 ml of d = 1.006 g/ml KBr in a Beckman SW41 centrifuge tube. After centrifugation at 37,000 rpm for 18 h at 15°C, VLDL and the other lipoproteins were collected from the top into 12 one-ml fractions. ApoB100 in individual fractions or pooled fractions was immunoprecipitated, resolved by SDS-PAGE, and visualized using a phosphorImager (Trio Typhoon; Amersham, GE Healthcare), with quantification by densitometry of the apoB100 bands.

### Statistical analyses

All experiments were conducted in triplicate at least two times. Data are typically expressed as the mean±SEM. Statistical differences were analyzed using GraphPad Prism software (GraphPad Software Inc., San Diego, CA) using an unpaired T test. Data in which more than two groups were compared were analyzed by ANOVA with post-hoc testing. A P value of less than 0.05 was considered significant and is indicated by *; *p*<0.01 is indicated by **, and *p*<0.001 by ***. Where displayed, NS denotes not significant.

## Results

### Roles of specific PI3-kinases in insulin-stimulated apoB100 degradation in primary hepatocytes

Mouse and rat primary hepatocytes secrete both apoB100 and apoB48-containing lipoproteins. In human liver, however, only apoB100 is secreted (and is exclusively associated with VLDL) because of the lack of the apoB mRNA editing activity that is required for apoB48 production. Given the greater relevance of apoB100 to human liver physiology, we wished to focus on this isoform. The major form of apoB in rodent liver is normally apoB48, and the sparseness of apoB100 hampers its accurate detection. To overcome this limitation, we turned to mice deficient in the apoB mRNA editing activity (*Apobec-1^−/−^*;[19]), in which mouse liver is “humanized” to produce only apoB100. Note that the effects of insulin on apoB100 are independent of the presence or the absence of hepatic apoB48 production (e.g., [7], [26], and data below).

**Figure 1:**
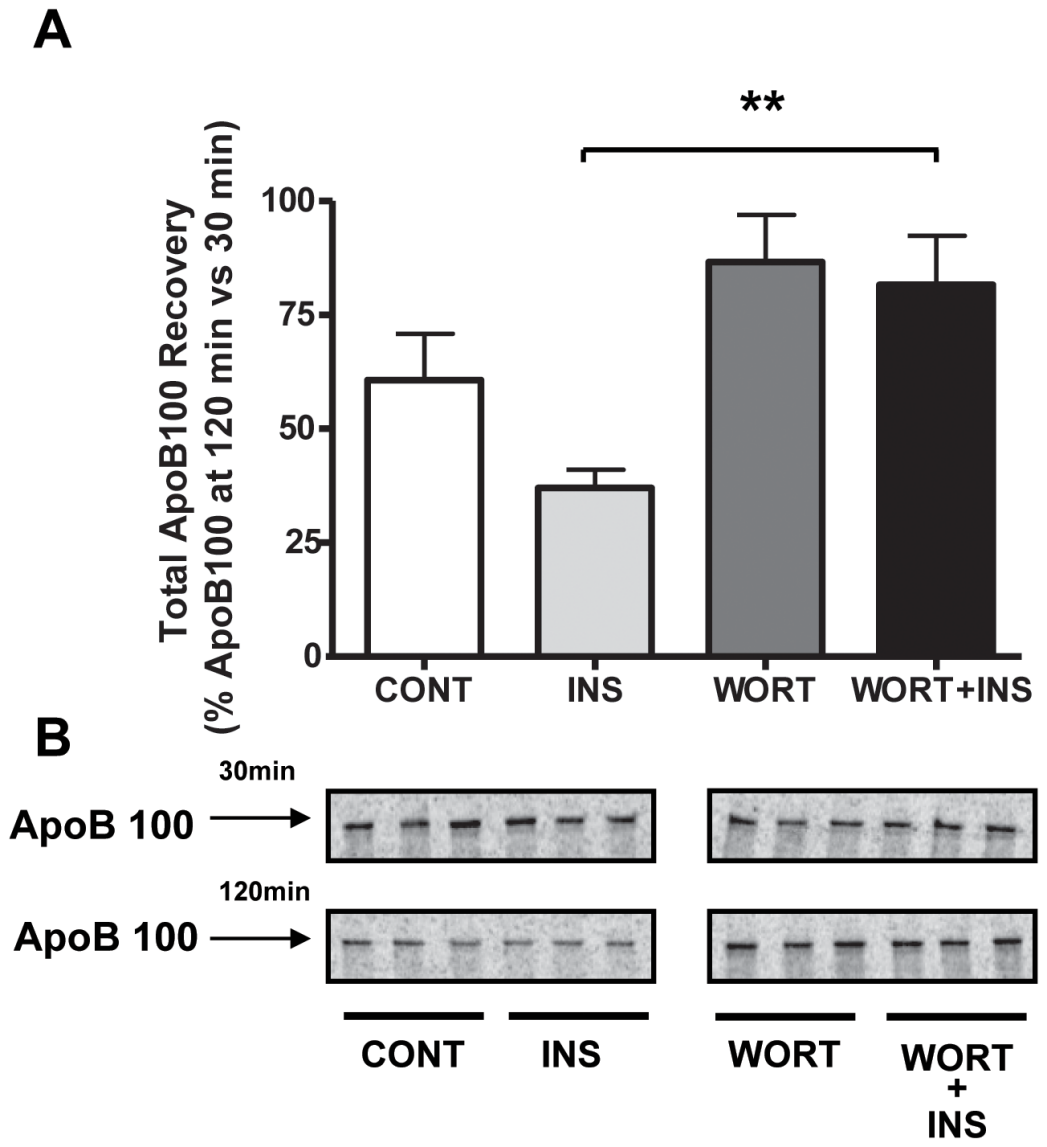
Insulin-stimulated apoB100 degradation in mouse primary hepatocytes is PI3-kinase- dependent. A) Primary hepatocytes from *Apobec1^−/−^* mice (which only synthesize apoB100) were incubated in media containing (INS) or lacking (CONT) insulin and/or wortmannin (WORT) and were pulse labeled for 15 min with [^35^S]-protein labeling mix and chased in non-radioactive medium for 30 and 120 min with the treatments maintained. ApoB100 was then immunoprecipitated and separated by SDS-PAGE and quantified as described in Materials and Methods. The histogram (mean±SEM) represents the results from 2 independent experiments, each performed in triplicate. B) Representative primary data of the experiments summarized in panel A; ** indicates P<0.01.

As shown in Figure 1A and 1B, we observed an increase in apoB100 degradation (reflected by a decrease in apoB100 recovery) in cells treated with insulin that was dependent on PI3-kinase activity, consistent with previous data [26]. Interestingly, when the cells were treated with insulin and the pan PI3-kinase inhibitor (wortmannin) or with wortmannin alone, apoB100 recovery rose. These combined results suggest that a PI3-kinase regulates a degradative process that is both insulin-stimulated and that occurs at a lower level during the basal turnover of apoB100.

**Figure 2:**
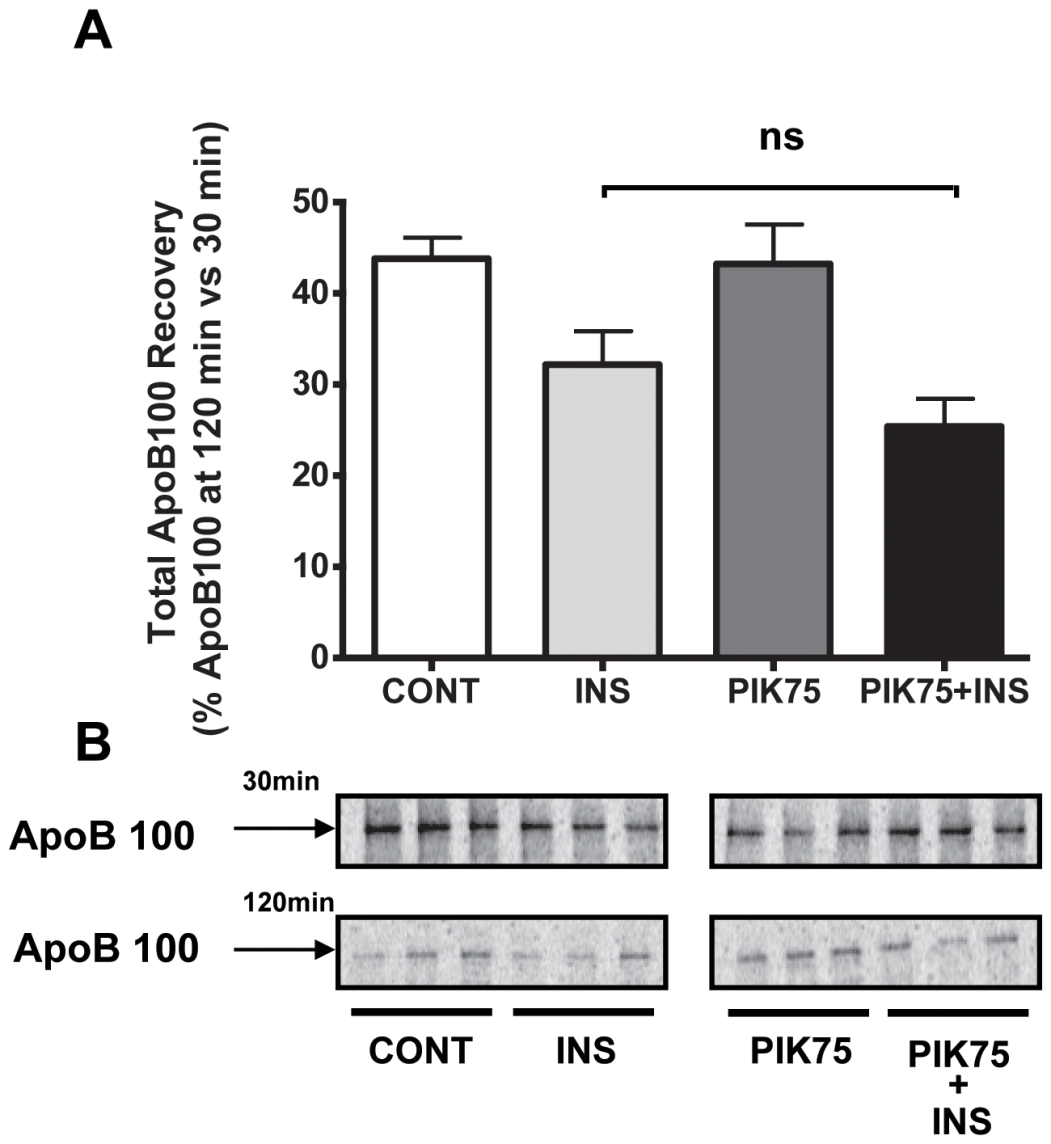
Class I PI3-kinase activity is dispensable for insulin-stimulated apoB100 degradation in mouse primary hepatocytes. A) Experiments were performed as in Figure 1, but in the presence or absence of the class I specific PI3-kinase inhibitor, PIK75. The histogram (mean±SEM) represents the results from 2 independent experiments, each performed in triplicate. B) Representative primary data of the experiments summarized in panel A.

Because wortmannin is a pan PI3-kinase inhibitor, we next wished to establish which specific enzyme class mediated these effects. Class III PI3K is not known to be a target of insulin signaling [15], so we considered the other two classes. We began by using an established class I-specific inhibitor, PIK75 [24]. As shown in Figure 2A and 2B, PIK75 did not prevent apoB100 degradation stimulated by insulin; rather, we observed a trend toward an enhancement of the insulin effect. Relevant to the autophagy studies, below, this result is consistent with the finding that class I PI-3 kinase activity is a negative regulator of autophagy [14].

**Figure 3:**
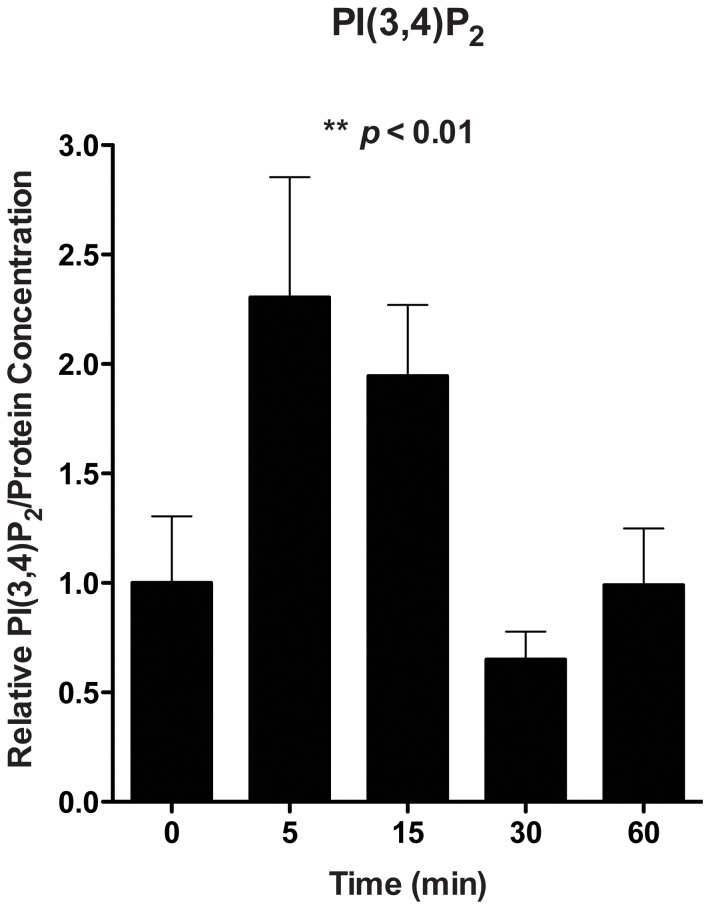
Insulin stimulates class II PI-3 kinase activity in mouse primary hepatocytes. Primary hepatocytes from *Apobec1^−/−^* mice were cultured in serum free conditions for 16 h before the addition of insulin to a final concentration of 100 nM. At the indicated times, the cells were harvested in ice cold 0.5 M TCA, and acidic lipids were extracted. PI(3,4)P_2_ concentrations were measured by dot blotting using anti-PI(3,4)P_2_ antibody (n = 3 at each time point). Data represent the mean±SEM.

Like the class I enzymes, class II PI3-kinases have been shown responsive to insulin stimuli [27], so we then assessed the role of class II PI3-kinase on apoB100 degradation after insulin addition. We first established that class II activity was stimulated by insulin by measuring the production of one of its specific products, PI(3,4)P_2_. As shown in Figure 3, we observed a strong and transient increase of PI(3,4)P_2_ upon insulin stimulation, as anticipated from published results [27].

**Figure 4:**
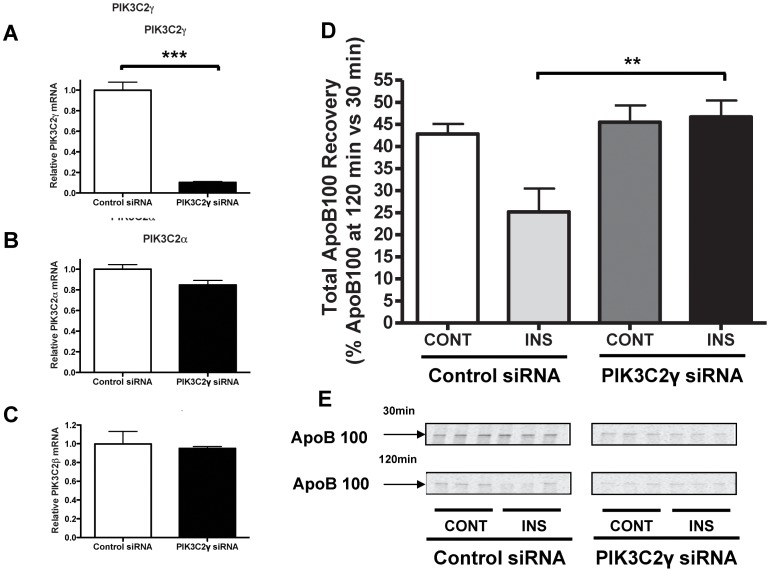
Insulin-stimulated apoB100 degradation in mouse primary hepatocytes is dependent on class II PI3-kinase gamma. Primary hepatocytes from *Apobec1^−/−^* mice were transfected with control (scrambled) siRNA or class II PI3-kinase (PIK3C2γ) specific siRNA. After a total of 48 h after transfection, (A) PIK3C2γ, (B) PIK3C2α, and (C) PIK3C2β mRNA levels were assessed by two-step qRT-PCR, and their abundance was normalized to 28S rRNA. The histogram (mean±SEM) represents the results from 2 independent experiments, each one performed in triplicate. D) Control or PIK3C2γ siRNA transfected primary hepatocytes from *Apobec1^−/−^* mice were incubated in medium with (INS) or without (CONT) insulin, pulse-labeled for 15 min with [^35^S]-protein labeling mix, and then chased for 30 and 120 min with the treatments maintained. Total apoB100 recovery and quantification were as in Figure 1. The histogram (mean±SEM) represents the results from 2 independent experiments, each one performed in triplicate; ** and *** indicate P<0.01 and 0.001, respectively. E. Representative primary data of the experiments summarized in panel D.

Due to the absence of a specific inhibitor of the class II PI3-kinase, we instead used siRNA to knock down the major PI3-kinase class II isoform present in the liver [28]–[30], PI3-kinase class II gamma, and examined the impact on the insulin response. The siRNA directed against this class II isoform suppressed its RNA levels by 90% (Figure 4A). Also, by using a functional assay to measure its specific product, PI(3,4)P_2_ after insulin stimulation, we observed nearly complete abolition of PI3-kinase class II gamma activity in the silenced hepatocytes (Figure S1). Even though the gamma isoform is highly related to the alpha and beta isoforms, the targeted siRNA was without a significant effect on their mRNA levels (Figure 4B and 4C), thus confirming the specificity of the siRNA. Notably, as shown in Figure 4D and E, insulin-stimulated apoB100 degradation was completely abrogated by knocking down the gamma isoform of class II PI-3 kinase, thereby demonstrating its key role in this process.

### Insulin-stimulated apoB100 degradation requires autophagy

Insulin stimulates a form of apoB100 degradation that is a pre-secretory, but a post-ER process [13]. We previously showed that omega-3 fatty acids (e.g., docosahexaenoic acid; DHA) stimulate apoB100 degradation [31], also by a post-ER, pre-secretory proteolysis process (PERPP; [9]. PERPP-mediated turnover of apoB100 was subsequently found to be through the autophagy pathway [10]. The broad similarities between insulin and DHA-stimulated apoB100 degradation raised the issue of whether the effect of insulin on apoB100 degradation also involved autophagy.

**Figure 5:**
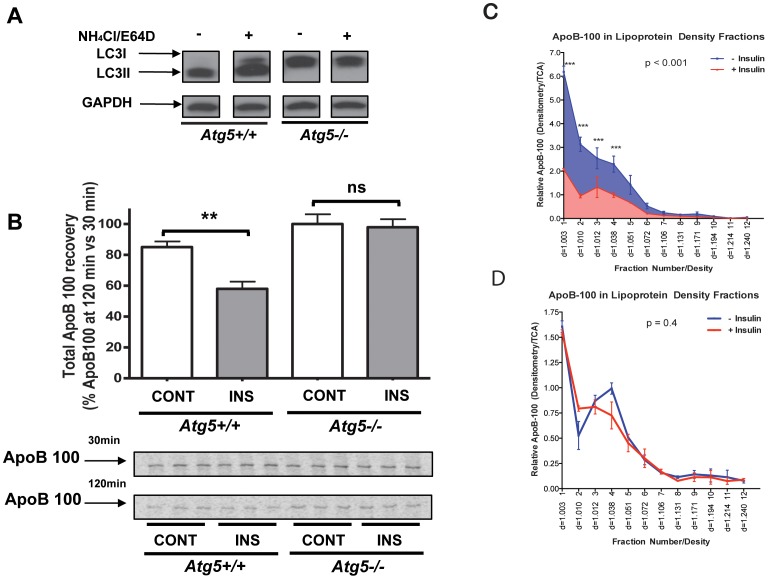
Effects of insulin on apoB100 degradation and VLDL-apoB100 secretion are blunted in autophagy-deficient mouse primary hepatocytes. Primary hepatocytes were isolated from mice with floxed alleles of *Atg5* (*Atg5^+/+^*) or *Atg5^f/f^ x Alb-Cre* (*Atg5^−/−^*) mice (i.e., mice with hepatic deficiency of Atg5), and cultured in serum-free conditions for 16 h before insulin addition. A) A western-blot for LC3 was performed with lysates of primary hepatocytes from *Atg5^+/+^* or *Atg5^−/−^* mice; “(+)” represents the condition in which lysosomal degradation has been blocked (20 mM NH_4_Cl + 10 µM E64D) to increase LC3 recovery and “(-)” represents untreated cells. When autophagy is active, LC3 (“LC3-I”) is lipidated to form LC3-II. GAPDH was used as the loading control. B) Primary hepatocytes from *Atg5^−/−^* or *Atg5^+/+^* mice were incubated in media with (INS) or without insulin (CONT) and pulse-labeled for 15 min with [^35^S]-protein labeling mix, and were then chased for 30 and 120 min in non-radioactive medium with the treatments maintained. Total apoB100 recovery and quantification were as in Figure 1. The histogram (mean±SEM) represents the results from 2 independent experiments, each one performed in triplicate; ** indicates P<0.01. C) Primary hepatocytes from *Apobec1^−/−^* mice were labeled with [^35^S]-protein labeling mix for 4 h in the presence (+ Insulin) or absence (− Insulin) of 100 nM insulin. Conditional media samples were collected and lipoproteins were separated by density gradient ultracentrifugation. ApoB100 in individual fractions was immunoprecipitated, resolved by SDS-PAGE, and quantified by densitometry after bands were detected by a phosphorImager. The statistical significance of the comparisons between the density profiles is based on 3 independent replicate experiments. D) An experiment similar to the one in panel C was performed, but using Atg5-deficent primary hepatocytes prepared from *Apobec1^−/−^* mice.

To directly test this hypothesis, experiments were conducted in primary hepatocytes from *Atg5*-deficient mice [22]. Atg5 participates in the early steps in the formation of autophagosomes [14]. To create mice with a liver-specific deficiency of Atg5 (designated as *Atg5^−/−^* in Figure 5), we crossed mice having floxed alleles of *Atg5* with *Alb-Cre* mice expressing Cre-recombinase driven by the albumin promoter [32]. *Atg5*-floxed mice were used as sources of control hepatocytes (*Atg5^+/+^* in Figure 5). Autophagic activity was examined in the control and liver-specific *Atg5*-deficient mice by assaying for the lipidation of the autophagosomal membrane protein, LC3 to the form called LC3-II, which is associated with autophagosomes [33]. As shown in Figure 5A, LC3-II was evident only in the control (*Atg5^+/+^*) hepatocytes, and the amount increased when lysosomal degradation was inhibited by NH_4_Cl and E64D (“+”). This increase was expected because after autophagosomes fuse with lysosomes, LC3-II is degraded. In contrast, only the LC3 precursor (LC3-I) was evident in the *Atg5^−/−^* hepatocytes, regardless of whether lysosomal degradation had been inhibited, indicating the lack of autophagosome formation. These data confirm that the autophagy pathway is inactive in the *Atg5^−/−^* hepatocytes. We then assessed the impact of insulin-stimulated apoB100 degradation in these cells. In the *Atg5^+/+^* hepatocytes there was the expected decrease in apoB100 recovery from insulin-treated cells (Figure 5B). In contrast, in the *Atg5*-deficient hepatocytes, insulin treatment had no effect on apoB100 recovery.

Independent confirmation for a role for autophagy in insulin-stimulated apoB100 degradation came from studies of class III PI3-kinase, which as noted earlier, is not a target of insulin [15]. Class III PI3-kinase (also called Vps34 or PIK3C3), however, plays a critical general role in autophagy in most cell types [14], including liver [34]. Therefore, if autophagy were involved in insulin-stimulated apoB100 degradation, silencing of Vps34 should have an effect on this process. This expectation was confirmed by pulse-chase studies after siRNA knock down of Vps34 (Figure S2). To establish that the loss of insulin-stimulated degradation of apoB100 was associated with a general effect of the Vps34 knock down on autophagy, we assessed the level of general autophagic activity by measuring (by western blot) the active, lipidated form of the autophagosome protein LC3 (LC3-II; [33]). As shown in Figure S2, knock-down of Vps34, but not of PI3-kinase class II gamma, increased the level of LC3-II, reflecting a reduction in general autophagic activity [34].

Insulin-stimulated apoB100 effects have been reported to be preferential for apoB100 normally associated with the more lipidated particles (e.g., VLDL [35]). Therefore, we performed a gradient density analysis of lipoproteins secreted from primary hepatocytes of *Apobec1^−/−^* mice that had been incubated in the presence or absence of insulin. As expected, we observed that insulin treatment decreased the recovery of apoB100 normally associated with the largest (highly lipidated) particles (density fraction 1.003; Figure 5C). In contrast, the effect of insulin treatment on apoB100 depletion in the density fractions normally containing these highly lipidated particles was lost in *Apobec1^−/−^* hepatocytes deficient in Atg5 (Figure 5D). Overall, these data strongly suggest that apoB100 normally associated with highly lipidated apoB-lipoproteins, including VLDL, is targeted for insulin-stimulated degradation via the autophagic pathway.

## Discussion

There are two major findings in this report on the mechanisms underlying insulin-stimulated degradation of apoB100, namely, that the process 1) requires autophagy, and, 2) depends on the gamma isoform of the class II PI3-kinase. In addition, we provide evidence that autophagy also contributes to basal apoB100 turnover, for which other classes of PI3-kinases may also play a role.

We and others previously reported that autophagy is critical during other examples of induced apoB100 post-ER, pre-secretory proteolysis (PERPP). Notably, PERPP mediates the degradation of apoB100 in the presence of fish oils or glucosamine [9], [12], or when the apolipoprotein contains a specific structural mutation [11]. In these cases, there is sufficient lipidation for pre-VLDL particles to form, but there is aberrant post-ER maturation to fully lipidate VLDL. That autophagy is an important disposal process for apoB100 in these examples is consistent with at least two lines of reasoning. First, VLDL assembly is a prolonged process that begins in the ER and continues post-ER through the intermediate compartment and in the Golgi (reviewed in [36]–[38]). Thus, unlike the majority of secretory proteins in which quality control is exerted primarily at the ER level [39], [40], the maturation of apoB100-containing particles is subject to multiple checkpoints, including important steps beyond the ER. Second, the autophagic process is specialized to degrade substrates of enormous size, including whole organelles [14], and so it makes sense that incompletely formed or abnormal VLDL particles (e.g., containing aggregated apoB100; [10]) will need to employ a disposal route that can accommodate substrates far bigger than typical secretory proteins.

Despite the expanding evidence for autophagy being a final common pathway for the degradation of apoB100 that has progressed past the ER, it remains mysterious how apoB100 or its associated lipoprotein particles are identified for disposal. During autophagy, the molecular features underlying how specific substrates are recognized are poorly defined [41]. As implied above, we favor a scenario in which a failure to fully lipidate pre-VLDL to the mature form in a post-ER compartment targets the intermediate particles for autophagy. This is based on the finding that during the fish oil and insulin-stimulated autophagic degradation of apoB100, as well as the autophagic destruction of A31P mutant apoB100, pre-VLDL particles are formed and reach the Golgi, but VLDL maturation is impaired (V. Maitin, U. Andreo, E. Fisher, unpublished results, [35], and [11], respectively).

Another novel aspect of this study is the involvement of class II PI3-kinase in insulin-stimulated apoB100 degradation, which appears to be the first link of this class of PI3-kinases to autophagy. We were led to investigate PI3-kinases because the pan-PI3-kinase inhibitor wortmannin had been shown to block insulin-stimulated apoB100 degradation (e.g., [13] [15]), as we also found (Figure 1). As noted earlier, there are three major classes of PI3-kinases (see [16] for a recent review). Class I contains the isoforms associated with insulin-stimulated regulation of glucose metabolism, and has been historically the pathway of interest in diabetes research. Evidence against this class mediating insulin-stimulated apoB100 degradation, however, is that the signaling cascade initiated by class I PI3-kinase inhibits autophagy through Akt-dependent activation of mTOR [14]. Furthermore, it was shown that insulin-mediated apoB100 degradation was Akt-independent [42]. Experimental confirmation for the lack of involvement of class I PI3-kinase in insulin-stimulated apoB100 degradation was provided in the present study (Figure 2). Specifically, we found that a class I-specific inhibitor did not block the insulin effect; instead, apoB100 recovery tended to be decreased, most likely through diminished mTOR activation, which resulted in increased autophagy.

There is firm evidence that the action of class III PI3-kinase increases general autophagy in most tissues (e.g., see [14]), including liver [34]. Consistent with a role for autophagy in insulin-stimulated apoB100 degradation, then, was our finding that knocking down class III PI3-kinase prevented degradation (Figure S2). The current understanding is that the product of the PI3-kinase enzymatic reaction, PI(3)P, serves to promote the formation of membrane-bound complexes that include the key autophagy initiating factor beclin-1 [16]. There is no evidence, however, that the activity of class III PI3-kinase is stimulated by insulin [15], [16]. In contrast, class II PI3-kinases are known to be phosphorylated in response to insulin and other growth factors [27]. There are three sub-classes of the class II isoform, namely alpha, beta, and gamma. Gamma is the most abundant one in the liver [28]–[30], explaining why we chose to manipulate the expression of this isoform in the present studies.

Compared to classes I and III, relatively little is known about the biological effects of the class II isoform. It is enriched in the juxtanuclear Golgi in rat liver [29], and a proposed role for its activity is to regulate vesicle traffic from the trans-Golgi network [28]. Given that the insulin-stimulated effect on apoB100 degradation requires post-ER PI3-kinase dependent trafficking [13], and that we have shown that fish oil-stimulated autophagy requires trafficking from the Golgi [10], it is tempting to speculate that a class II isoform mediates these transport events. It is unlikely that insulin signaling through class II PI3-kinase is operating through induction of Atg5 itself because in autophagy-competent hepatocytes the level of Atg5 was unaltered after insulin stimulation (L. Guo, E. Fisher, data not shown). Obviously, further investigation will be needed to evaluate in depth the many possible mechanisms underlying the class II PI3-kinase effects on insulin-stimulated apoB100 degradation.

In conclusion, the present results indicate that autophagy and class II PI3-kinase are required for the degradation of apoB100 in insulin-stimulated hepatocytes. In insulin-resistant people, apoB100 and VLDL are overproduced (e.g., [43], [44]). Consequently, our results suggest that this may represent the loss of the insulin-stimulated autophagy-dependent pathway for apoB100. This intriguing possibility can be indirectly tested by extending our studies to insulin-resistant mice competent or deficient in autophagy or class II PI3-kinase activity.

## Supporting Information

Figure S1 **Effect of siRNA knockdown of PI3K class II gamma on the insulin-stimulated production of its specific product PI(3,4)P_2_.** Levels of PI(3,4)P_2_ were assessed as in Figure 3 in primary hepatocytes (isolated from *Apobec1^−/−^* mice) treated with either control or class II PI3-kinase gamma siRNA.
(TIF)

Figure S2 **Class III PI3-kinase knockdown reduces insulin-stimulated apoB100 degradation and autophagic activity in mouse primary hepatocytes.** A) Primary hepatocytes (isolated from *Apobec1^−/−^* mice) were treated with either control or Class III PI3-kinase (Vps34) siRNA. 48 h after siRNA transfection, pulse-chase experiments were performed. ApoB100 recovery results are represented in the histogram (mean±SEM) from two independent experiments with each performed in triplicate. B) Western blotting analysis of LC3-II (normalized to GAPDH) in primary hepatocytes transfected with control, PIK3C2gamma or PIK3C3 (Vps34) siRNA as in panel A.
(TIF)
