# Supplementary figures and images for: Insulin-Stimulated Degradation of Apolipoprotein B100: Roles of Class II Phosphatidylinositol-3-Kinase and Autophagy
Source: PLoS One. 2013 Mar 13;8(3):e57590. doi: 10.1371/journal.pone.0057590 (PMC3596368; doi:10.1371/journal.pone.0057590)

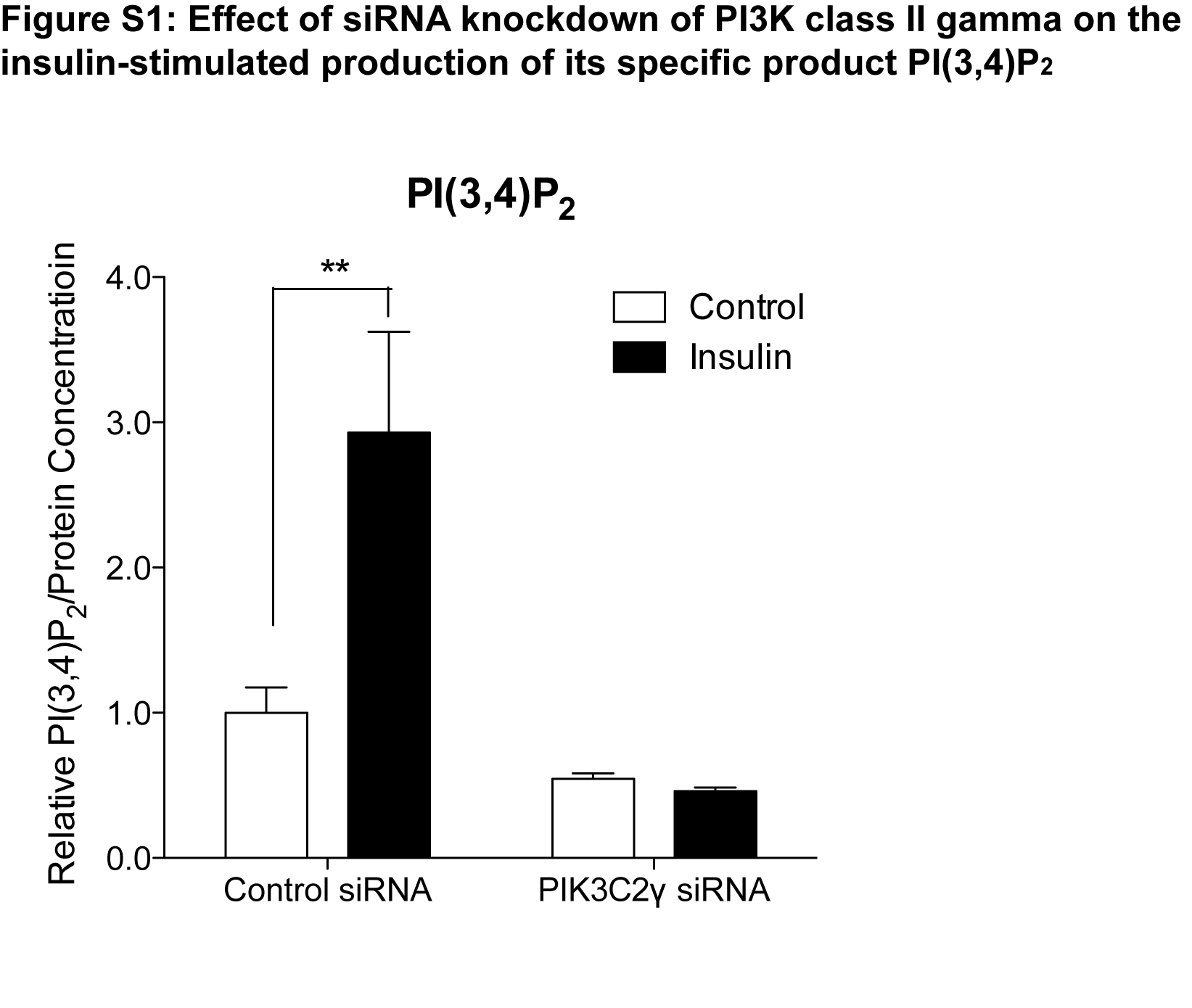

Supplement: Figure S1 — Effect of siRNA knockdown of PI3K class II gamma on the insulin-stimulated production of its specific product PI(3,4)P2. Levels of PI(3,4)P2 were assessed as in Figure 3 in primary hepatocytes (isolated from Apobec1−/− mice) treated with either control or class II PI3-kinase gamma siRNA. (TIF) [file pone.0057590.s001.tif]

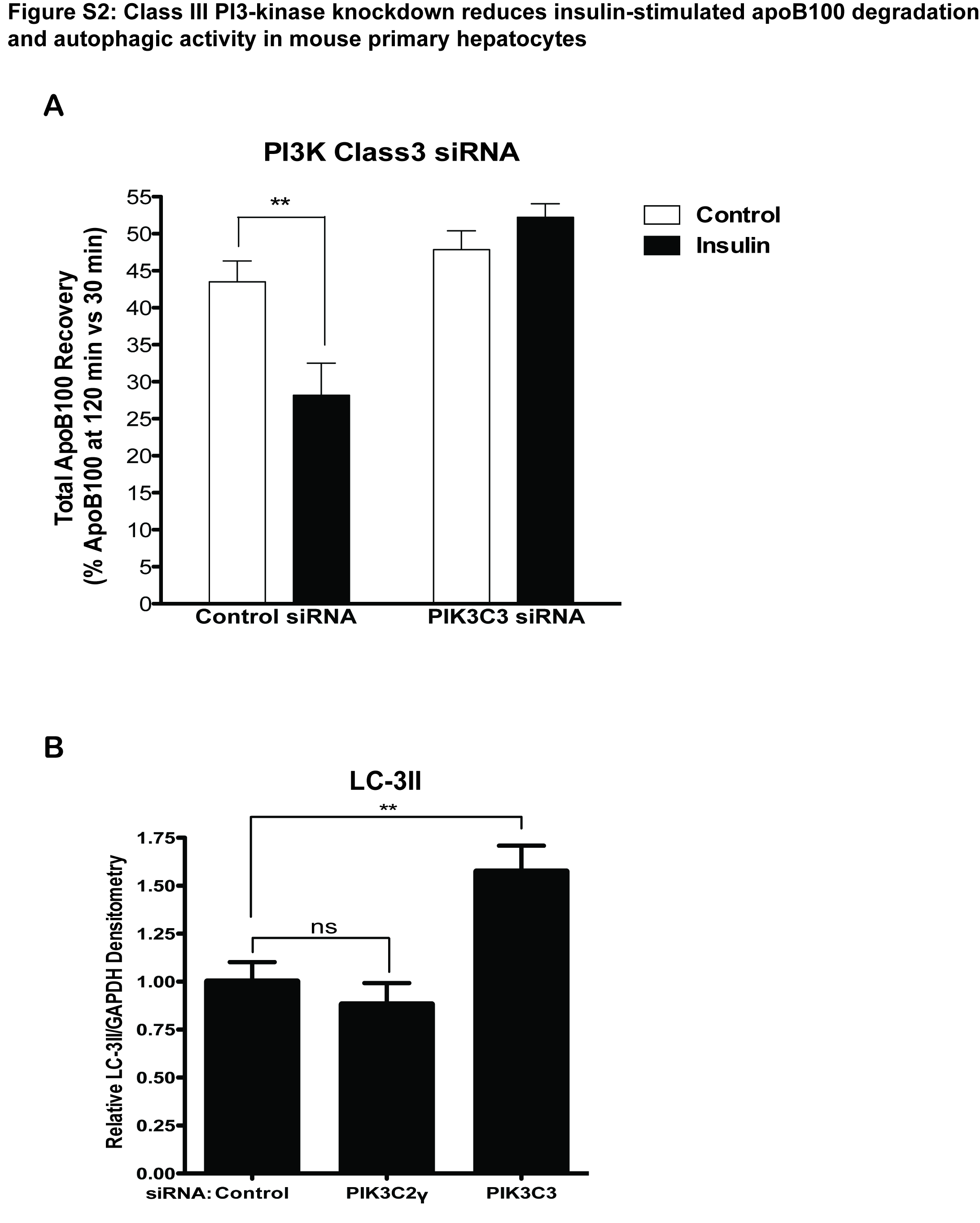

Supplement: Figure S2 — Class III PI3-kinase knockdown reduces insulin-stimulated apoB100 degradation and autophagic activity in mouse primary hepatocytes. A) Primary hepatocytes (isolated from Apobec1−/− mice) were treated with either control or Class III PI3-kinase (Vps34) siRNA. 48 h after siRNA transfection, pulse-chase experiments were performed. ApoB100 recovery results are represented in the histogram (mean±SEM) from two independent experiments with each performed in triplicate. B) Western blotting analysis of LC3-II (normalized to GAPDH) in primary hepatocytes transfected with control, PIK3C2gamma or PIK3C3 (Vps34) siRNA as in panel A. (TIF) [file pone.0057590.s002.tif]
